# Robust Identification of Noncoding RNA from Transcriptomes Requires Phylogenetically-Informed Sampling

**DOI:** 10.1371/journal.pcbi.1003907

**Published:** 2014-10-30

**Authors:** Stinus Lindgreen, Sinan Uğur Umu, Alicia Sook-Wei Lai, Hisham Eldai, Wenting Liu, Stephanie McGimpsey, Nicole E. Wheeler, Patrick J. Biggs, Nick R. Thomson, Lars Barquist, Anthony M. Poole, Paul P. Gardner

**Affiliations:** 1 Department of Biology, University of Copenhagen, Copenhagen, Denmark; 2 School of Biological Sciences, University of Canterbury, Christchurch, New Zealand; 3 Biomolecular Interaction Centre, University of Canterbury, Christchurch, New Zealand; 4 Institute of Veterinary, Animal & Biomedical Sciences, Massey University, Palmerston North, New Zealand; 5 Allan Wilson Centre for Molecular Ecology & Evolution, Massey University, Palmerston North, New Zealand; 6 Pathogen Genetics, Wellcome Trust Sanger Institute, Hinxton, United Kingdom; 7 Institute for Molecular Infection Biology, University of Wuerzburg, Wuerzburg, Germany; Rutgers University, United States of America

## Abstract

Noncoding RNAs are integral to a wide range of biological processes, including translation, gene regulation, host-pathogen interactions and environmental sensing. While genomics is now a mature field, our capacity to identify noncoding RNA elements in bacterial and archaeal genomes is hampered by the difficulty of *de novo* identification. The emergence of new technologies for characterizing transcriptome outputs, notably RNA-seq, are improving noncoding RNA identification and expression quantification. However, a major challenge is to robustly distinguish functional outputs from transcriptional noise. To establish whether annotation of existing transcriptome data has effectively captured all functional outputs, we analysed over 400 publicly available RNA-seq datasets spanning 37 different Archaea and Bacteria. Using comparative tools, we identify close to a thousand highly-expressed candidate noncoding RNAs. However, our analyses reveal that capacity to identify noncoding RNA outputs is strongly dependent on phylogenetic sampling. Surprisingly, and in stark contrast to protein-coding genes, the phylogenetic window for effective use of comparative methods is perversely narrow: aggregating public datasets only produced one phylogenetic cluster where these tools could be used to robustly separate unannotated noncoding RNAs from a null hypothesis of transcriptional noise. Our results show that for the full potential of transcriptomics data to be realized, a change in experimental design is paramount: effective transcriptomics requires phylogeny-aware sampling.

## Author Summary

We have analysed more than 400 public transcriptomes, generated using RNA-seq, from almost 40 strains of Bacteria and Archaea. We discovered that the capacity to identify noncoding RNA outputs from this data is strongly dependent on phylogenetic sampling. Our results show that, for the full potential of transcriptomics data as a discovery tool to be realized, a change in experimental design is critical: effective comparative transcriptomics requires phylogeny-aware sampling. We also examined how comparative transcriptomics experiments can be used to effectively identify RNA elements. We find that, for RNA element discovery, a phylogeny-informed sampling approach is more effective than analyses of individual species. Phylogeny-informed sampling reveals a narrow ‘Goldilocks Zone’ (where species are not too similar and not too divergent) for RNA identification using clusters of related species. In stark contrast to protein-coding genes, not only is the phylogenetic window for the effective use of comparative methods for noncoding RNA identification perversely narrow, but few existing datasets sit within this Goldilocks Zone: by aggregating public datasets, we were only able to create one phylogenetic cluster where comparative tools could be used to confidently separate unannotated noncoding RNAs from transcriptional noise.

## Introduction

Genome sequencing has transformed microbiology, offering unprecedented insight into the physiology, biochemistry, and genetics of Bacteria and Archaea [1]–[4]. Equally, careful examination of transcriptional outputs has revealed that bacterial and archaeal transcriptomes are remarkably complex [5]. Roles for RNA include regulation, post-transcriptional modification and genome defense processes [6]–[10]. However, our view of the microbial RNA world still derives from a narrow sampling of microbial diversity [11]. Additional bias comes from the fact that many microbes are not readily culturable [12]. The development of metagenomics and initiatives such as the Genomic Encyclopedia of Bacteria and Archaea (GEBA) project have sought to redress these biases, generating genomes spanning undersampled regions of the bacterial and archaeal phylogeny [1], and sequencing uncultured or unculturable species through metagenomics [2], [13]–[16].

A further source of bias in our genome-informed view of microbes derives from a protein-centric approach to genome annotation. The majority of genome sequences deposited in public databases carry limited annotation of noncoding RNAs and cis-regulatory elements, yet it is rapidly becoming clear that RNA is essential to our understanding of molecular functioning in microbes [17].

The paucity of annotations is understandable, as RNA gene annotation is non-trivial [18], [19]. However, the increasing number of roles for RNAs uncovered through experimental and bioinformatic studies make illuminating this “dark matter” all the more urgent. Among the remarkable discoveries made are: riboswitch-mediated regulation [9], [20], transcriptional termination by RNA elements [21]–[23], identification of novel natural catalytic RNAs [24]–[27], CRISPR-mediated acquired immunity [28], [29], temperature-dependent gene regulation [30], [31], and sno-like RNAs in Archaea [32]–[34]. The Rfam database [22], [35] provides a valuable platform for collating and characterising these and other families of noncoding RNA. However, a recent comparative analysis [36] revealed that fewer than 7% of RNA families within Bacteria and less than 19% in Archaea show a broad phylogenetic distribution (that is, presence in at least 50% of sequenced phyla). Crucially, that analysis revealed that underlying genome sequencing biases were a major contributor to this pattern, and that the wider genomic sampling provided by the GEBA dataset [1] did help improve identification of broadly-conserved RNA families [36]. Tools such as RNA-seq [37] and transposon insertion sequencing [38]–[40] promise to complement comparative genomics tools for RNA family discovery, and it may be possible to use a mix of data types in the identification of RNA elements. However, to date, no systematic analysis of available data has been undertaken, suggesting ncRNAs may be hidden in the deluge of published data.

We have therefore assessed the value of RNA-seq data for identification of unannotated non-coding and cis-regulatory RNA elements in bacterial and archaeal genomes. We show that numerous, hitherto uncharacterised, expressed RNA families are lurking in publicly available RNA-seq datasets. We find that poor sequence conservation for RNA families limits the capacity to identify evolutionarily conserved, expressed ncRNAs from existing genomic and transcriptomic data. Our results suggest that maximising phylogenetic distance, a sampling strategy effective for identification of novel protein families [1], [2], is not the most effective strategy for ncRNA identification. Instead, our results show that, for RNA element identification, sequencing clusters of related microbes will generate the greatest benefit.

## Results

### Non-coding RNA elements dominate bacterial and archaeal transcriptional profiles

To assess the relative contribution of noncoding RNAs and protein-coding genes to transcriptional output, we collected all publicly-available bacterial and archaeal RNA-seq datasets (available as of August 2013), spanning 37 species/strains and 413 datasets. For all datasets, we supplemented publicly available genome annotations with screening for additional loci against the Pfam and Rfam databases [22], [35], [41], [42], followed by manual identification of expressed unannotated regions that have previously been dubbed RNAs of Unknown Function (RUFs) [43]. This latter annotation yielded 922 expressed RUFs.

**Figure 1:**
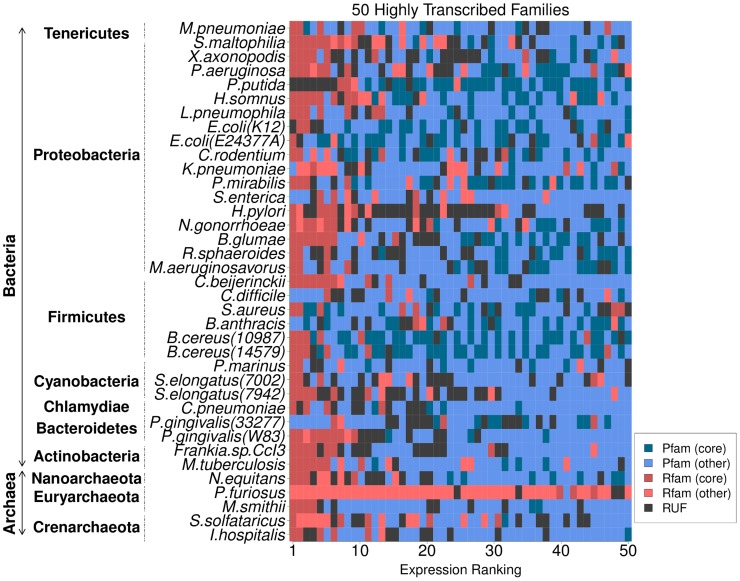
Identification of transcribed elements across publicly-available RNA-seq data. Non-coding RNA elements show high expression across transcriptomes. Both annotated Rfam families (red - core Rfam families (see Methods) are dark red, all others are light red) and expressed RUFs (black) are among the highest expressed outputs in transcriptomes (blue - core Pfam families (see Methods) are dark blue, all others are light blue). For each strain we generated relative rankings of expression spanning protein coding genes, RNA genes and candidate RUFs. Accurately estimating expression levels from read depths is confounded by a number of factors (e.g. sample preparation, overall sequencing depths, rRNA depletion, etc.). For consistency, we have ranked genes for each strain and compared rankings instead of comparing the read depths directly between strains. For a given strain, the annotated genes were ranked based on the median read depth of the annotated region. RUFs were manually picked by masking out annotated genes and selecting regions showing evidence of expression by inspecting read depth across the genome. This yielded 844 gene candidate sequences in Bacteria and 78 in Archaea. The plot contains the 50 most highly expressed elements for each strain/species.

We next examined the relative abundance of transcripts within each RNA-seq dataset, yielding an expression rank for individual transcripts. This analysis reveals that most transcriptomes are dominated by highly expressed non-coding RNA outputs (Figure 1) (P-value 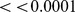, Chi-square test of observed vs. expected ratios and Fisher's Exact test on the counts). In addition to well-characterised RNAs (rRNA, tRNA, tmRNA, RNase P RNA, SRP RNA, 6S and sno-like sRNAs), and known cis-regulatory elements (riboswitches, leaders and thermosensors - Table S1), the top 50 most abundant transcriptional outputs (Figure 1) across the 32 Bacteria and 5 Archaea in our dataset included a total of 308 RUFs.

### Comparative analyses reveal that highly expressed transcripts are often poorly conserved

**Figure 2:**
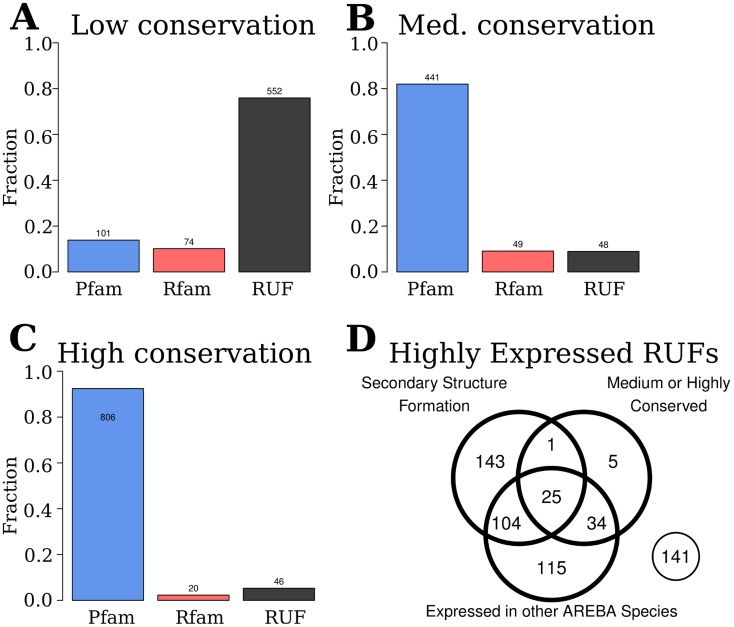
Many ncRNAs and RUFs are highly expressed but show limited conservation across represented strains/species. **A–C:** We have defined the “family conservation” for Pfam, Rfam and RUFs based upon the maximum phylogenetic distance (using structural SSU rRNA alignments) between any two strains hosting the family. We have divided the highly expressed transcripts (ranks 1–204) into Low, Medium and High conservation groups based on the lower-quartile, inter-quartile range and the upper-quartile of the family conservation measure (see Methods for further details). Both the known Rfam families and the RUFs identified in this analysis are often highly expressed transcripts. In contrast to protein-coding transcripts (blue), where highly-expressed transcripts are well-conserved, the opposite is true of many non-coding RNA elements (Rfam, red; RUFs, black). Notably, the greatest proportion of highly expressed Rfam-annotated RNA elements show a narrow evolutionary distribution. This is also reflected in the RUFs identified in this study. **D:** Venn diagram of the 568 highly expressed RUFs. Each RUF was analysed to look for evidence of secondary structure formation, level of conservation, and evidence of expression in at least one other RNA-seq dataset. All RUFs showing expression in other strains/species are conserved in at least two strains/species, so the figure also shows that 219 highly expressed RUFs are conserved across a limited phylogenetic distance only.

To assess whether highly expressed RUFs possess features commonly associated with function, we employed three criteria: 1) evolutionary conservation, 2) conservation of secondary structure, 3) evidence of expression in more than one RNA-seq dataset. For this analysis, we compared and ranked transcriptional outputs across species/strains (see Methods for details). Based on the relative rank across RNA-seq datasets and the maximum phylogenetic distance observed across all genomes, each transcript was classified as high, medium or low expression, and high, medium or low conservation. This yielded a set of highly expressed transcripts consisting of 162 Rfam families, 568 RUFs and 1429 Pfam families. As expected [44]–[46], conserved, highly expressed outputs are dominated by protein-coding transcripts (Figure 2B&C). In contrast, transcripts that are highly expressed but poorly conserved are primarily RUFs (Figure 2A). Of the 568 RUFs identified, only 25 are supported by all three conservative criteria (conservation, secondary structure and expression) (Figure 2D), a further 138 RUFs are supported by two criteria (Figure 2D). Consequently, on these criteria, the vast majority of RUFs appear indistinguishable from transcriptional noise. However, as these RUFs are among the most highly expressed transcripts in public RNA-seq data, we next considered whether our criteria were sufficiently discriminatory to identify functional RNAs. It is well established that not all functional RNAs exhibit conserved secondary structure – antisense base pairing with a target is common, and does not require intramolecular folding [47]. This indicates that criterion 2 will apply to some, but not all functional RNA elements. Criteria 1 and 3 both derive from comparative analysis: criterion 1 requires an expressed RUF to be conserved in some other genome, while criterion 2 requires an expressed RUF to be expressed in another of the datasets in our study. We therefore sought to examine how effective our comparative analyses are given that the available data represent a small sample (transcriptomes from 37 strains) and given that biases in genome sampling across bacterial and archaeal diversity impact comparative analysis of RNAs [36].

### Comparative analysis reveals a ‘Goldilocks Zone’ for ncRNA identification

Effective comparative analysis requires appropriate phylogenetic distances between species under investigation [48]. For discovery of protein-coding gene families, maximising phylogenetic diversity across the tree of life has proven very effective [1], [2]. For non-coding RNA, underlying biases in genome sampling do affect the assessment of ncRNA conservation, and adding phylogenetic diversity improves the identification of broadly conserved ncRNA families [36]. However, few ncRNAs appear conserved across broad evolutionary distances [36]. We have therefore considered how species selection impacts comparative analysis as a tool for the identification of conserved ncRNAs.

To assess the effect of strain selection on our capacity to identify RNA families using comparative analysis, we first generated F84 phylogenetic distances between 2562 bacterial strains and 154 archaeal strains using SSU rRNA sequences from each strain (see Methods for details). Next, for each Rfam RNA family and Pfam protein family, we identified the maximum phylogenetic distance between any two species/strains that encode a given family. We then calculated the fraction of conserved RNA and protein families for a given phylogenetic distance.

**Figure 3:**
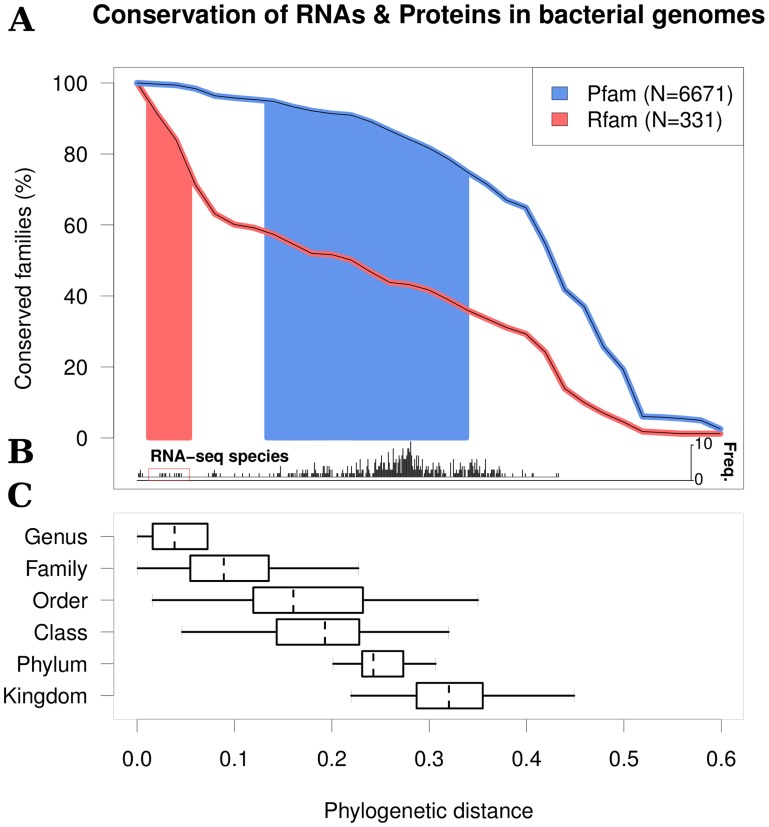
Conservation of protein and RNA families. All of the available full length Bacterial and Archaeal genomes were annotated using Rfam and Pfam models. For each Pfam/Rfam family, RNA-seq species or taxonomic group the “phylogenetic distance” is calculated using the maximum SSU rRNA F84 distance (see Methods for details). **A.** For the Pfam and the Rfam families we compare the levels of conservation as a function of phylogenetic distance using annotations of 2,562 bacterial genomes. E.g. 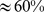 of RNA families are conserved between species from the same family, whereas 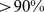 of protein families are conserved within the same taxonomic range. **B.** The barplot shows the distribution of all pairwise distances between the RNA-seq datasets. Eleven pairs (boxed) are in the Goldilocks Zone (See Figure 4 for further analysis). **C.** The ranges of phylogenetic distances for comparing species from different taxonomic groups.

This reveals a dramatic difference in evolutionary conservation of Rfam and Pfam families (Figure 3). While 80% of protein families are still conserved at the broad evolutionary distances that separate Bacteria and Archaea, the phylogenetic distance at which 80% of RNA families are conserved lies somewhere between the taxonomic levels of genus and family (Figure 3). The explanation for this rapid decay of RNA family conservation across long evolutionary time-scales is likely to be a combination of the limited abilities of existing bioinformatic tools to correctly align RNA sequences [49] and rapid turnover of non-coding RNAs during evolution [36].

These results in turn indicate that appropriate evolutionary distances for optimal comparative analysis differ greatly for protein- and RNA-coding genes. Figure 3 confirms the utility of the GEBA sampling strategy [1], [2] for protein-coding gene identification, since maximising phylogenetic diversity permits effective identification of conserved protein-coding genes. In contrast, at the largest phylogenetic distances, less than 40% of the RNA families are amenable to comparative analysis. These results define a ‘Goldilocks Zone’ (an evolutionary distance neither too close nor too distant) for ncRNA analysis through comparative analysis.

**Figure 4:**
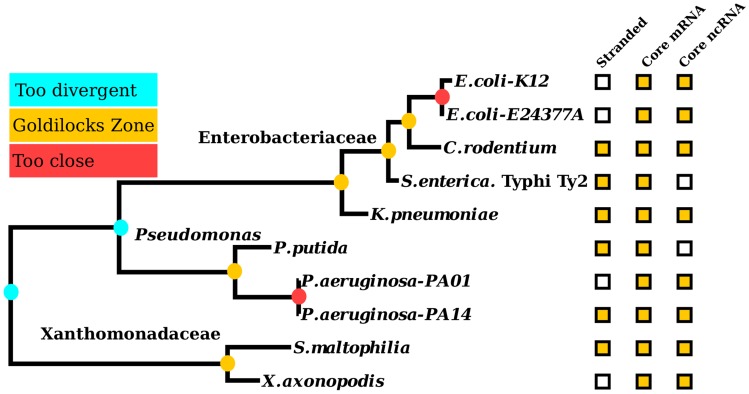
Public RNA-seq datasets that lie in the Goldilocks Zone. Ten strains with corresponding, publicly available RNA-seq data and phylogenetic distances in the Goldilocks Zone (Figure 3) have been identified. The maximum likelihood tree from a SSU rRNA alignment shows the relationships between these taxa. They fall into three clades, containing members of the families: Enterobacteriaceae and Xanthomonadaceae, and the genus: *Pseudomonas*. The nodes connecting taxa within the Goldilocks Zone are coloured gold, taxa that are too close are coloured red and those that are too divergent are coloured cyan. Each strain is annotated with gold boxes where there was stranded information, or if the majority of core mRNAs and ncRNAs (see Methods) were expressed (see Table S3 for the raw data).

In order to assess the potential for existing RNA-seq data to be used for ncRNA analysis, we mapped the pairwise distances between strains covered by the RNA-seq datasets in this study. Of the 506 possible pairs (excluding Bacteria vs Archaea), only 11 are in the Goldilocks Zone for RNA (phylogenetic distance between 0.0118 and 0.0542) covering 9 species/strains. While five pairs of datasets are ‘too hot’ (i.e. too close phylogenetically), the remaining 490 comparisons are ‘too cold’ for effective comparative RNA analysis (Figure 3). The datasets in the Goldilocks Zone span three distinct clades covering five Enterobacteria, three Pseudomanada, and two Xanthomonada (Figure 4).

**Figure 5:**
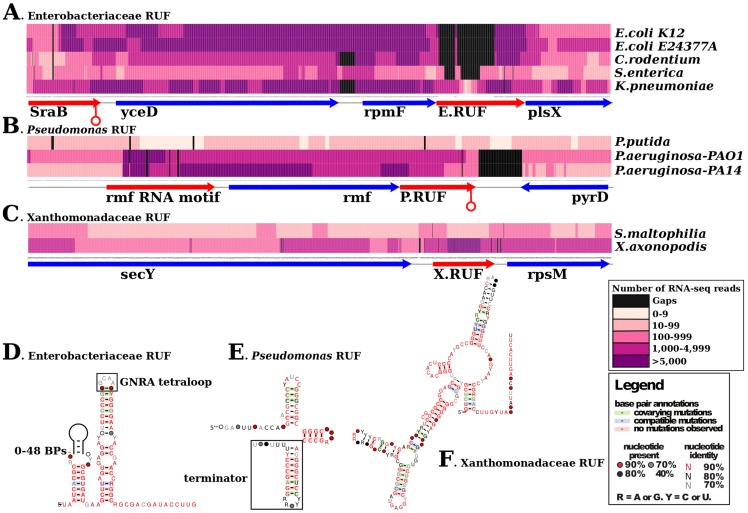
Comparative analysis of RNA-seq datasets in the Goldilocks Zone is a powerful approach for identifying RUFs. In this figure we illustrate data corresponding to 3 exemplar RUFs that show high covariation, conserved predicted secondary structures and are derived from one of the Goldilocks Zone clades shown in Figure 4. (**A–C**) The expression levels inferred from RNA-seq in the region encompassing each RUF. The regions contain a mix of ncRNAs (red arrows) and protein coding genes (blue arrows) and a RUF (red arrow). For each nucleotide, the total number of reads that map to that nucleotide was computed, and are presented as a heatmap; darker colours indicate high relative expression, lighter colours indicate low expression and black indicates a gap in the genomic alignment of the sequences for the locii. (**D–F**) R2R [68] representations of the predicted consensus secondary structures for exemplar RNAs of Unknown Function (RUFs) selected from the Enterobacteriaceae, *Pseudomonas* and Xanthomonadaceae data. Covariation is highlighted in green, structure-neutral variation is highlighted in blue, highly conserved regions are highlighted in pink. The Enterobacteriaceae RUF contains a conserved tetraloop of the GNRA or UNCG type, and there have been two independent insertions of hairpins in *S. enterica* and *K. pneumoniae* within the first hairpin. The *Pseudomonas* RUF hosts a 3′ rho independent transcription terminator.

We next calculated the percentage of conserved RUFs for all Enterobacterial strain pairs. On average, 83% of RUFs are conserved across the Goldilocks Zone. The two *E. coli* strains are extremely similar, and share 99% of their RUFs, suggesting that these strains are too similar for us to robustly separate expression of *bona fide* RNAs from noise. While these outputs could be genuine RNAs, these strains are in the ‘too hot’ region, meaning if everything is conserved, comparative power is lost. In contrast, only 12% of RUFs are conserved between strains/species pairs in the ‘too cold’ region (spanning clades; Figure 4) and of the 197 RUFs found through comparative analysis of transcriptomes within the Goldilocks Zone, only 19 show evidence of expression in another transcriptome outside of this zone. This suggests that the low number of RUFs from Figure 2D showing both conservation and expression is primarily a consequence of limited sampling. That said, mining RNA-seq data within the Goldilocks Zone permits a higher confidence in the identification of novel ncRNAs. Three examples of this are illustrated in Figure 5. These RUFs exhibit sequence and secondary structure conservation and are expressed at high levels across multiple Goldilocks Zone transcriptomes.

In summary, the Goldilocks Zone for RNA is surprisingly narrow, and suggests that optimal strain selection for RNA comparative analyses should comprise strains of the same species, members of the same genus, and closely related taxonomic families (Figure 3). Thus, the Goldilocks Zone for RNA is not encompassed by the sampling regimes currently being employed for protein family discovery.

## Discussion

Our analyses of over 400 publicly-available bacterial and archaeal RNA-seq datasets reveal that there is evidence for large numbers of RNAs of unknown function in public data. We find evidence for close to 1000 unannotated noncoding transcriptional outputs, but, given that RNA-seq experiments provide a snapshot of gene expression under specific experimental conditions, this number is likely to be far lower than the complete set of transcriptional outputs. Thus, the dataset we assembled for this project, which includes data generated by a number of labs and derives from various species and strains grown under a range of experimental conditions, is expected to represent a broad, though partial, census of total expression outputs across the species represented. Equally striking is the fact that, for the 922 RUFs identified in our study, over half (568) are among the most abundant transcripts. These results suggest that ncRNA may play an even greater role in the molecular workings of Bacteria and Archaea than hitherto realised.

This use of transcriptome data clearly improves our capacity to identify noncoding outputs: applying three criteria (sequence conservation, conservation of secondary structure, and expression in multiple strains/species) we have identified 163 high-confidence expressed RUFs from public data (Figure 2). An additional 405 RUFs are highly expressed across the transcriptomes we have examined, yet these do not show clear signs of sequence or structural conservation in other sequenced genomes. Given their high expression level, these seem unlikely to be transcriptional noise. Some may represent technical artefacts, but many could be *bona fide* lineage-specific ncRNAs with potentially novel functions.

Our results indicate that the greatest gain in analytical power for ncRNA discovery will come from phylogenetically-informed experimental design. Indeed, we find that this is critical to successful element identification, since the ‘Goldilocks Zone’ for optimal comparative analysis of RNA elements is surprisingly narrow. Hence, existing efforts to maximise phylogenetic coverage of genome space [1], [2] need to be complemented with fine-scale sampling of the tips (Figure 4). Indeed, analysing the few transcriptomes that span the Goldilocks Zone reveals a remarkable enrichment of transcripts showing evidence of structure, conservation and expression in other strains/species. Furthermore, it is worth noting that the RNA family conservation decays as the phylogenetic distance increases (shown in Figure 3). There is a possibility that the Rfam families used for this are biased. However, if a bias exists, it is towards families with higher conservation (as the families are constructed from published ncRNAs that are often discovered based upon sequence conservation [22], [35]). Thus, we might actually be overestimating RNA element conservation, making phylogenetically informed sampling even more important.

Given that isolation, cultivation and study of individual bacterial and archaeal strains can be extremely challenging [12] successful phylogeny-informed comparative RNA-seq will be a demanding endeavour, requiring complex sets of expertise spanning advanced culturing and isolation techniques, functional genomics capability and RNA bioinformatics. This places such a project beyond the reach of most individual labs. We therefore propose that comprehensive resolution of the comparative RNA-seq problem can best be resolved via a community-driven initiative: in recognition of the success of the GEBA project, we have dubbed this An RNA Encyclopedia of Bacteria and Archaea (AREBA). The appropriateness of this acronym will be especially clear to Japanophones, as, in Japanese, the phrase ‘areba’ (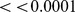) translates to ‘if there’.

## Materials and Methods

### Preprocessing and mapping

All available bacterial and archaeal genomes were downloaded from the European Nucleotide Archive (ENA) (2,562 and 154 genomes, respectively) [50]. RNA-seq datasets published as of August 2013 were collected, spanning 37 species/strains, 44 experiments and 413 lanes of sequencing data (Table S2). Most of these datasets were generated on the Illumina platform [51], with a few lanes from the SOLiD platform [52] and the 454 platform [53]. Where possible, FastQ files were downloaded, scanned for residual adapter sequences using AdapterRemoval (v1.5.4) [54], and mapped to the reference genome using Bowtie2 (v2.1.0) [55] for Illumina and 454 data and BFAST (v0.7.0a) [56] for SOLiD data.

### Producing consistent genome annotations

All genomes were re-annotated for both RNA genes and protein coding genes. Non-coding RNA genes were annotated using cmsearch (v.1rc4) [57] to identify homologs of RNA families from the Rfam database (v11.0) using the default “gathering threshold” (cmsearch –cut_ga) [22], [35]. Protein coding genes were annotated using three approaches: First, annotations were parsed from the ENA files. Secondly, Glimmer (v3.02) was run on all genomes to predict open reading frames (with parameters “-o7 -g45 -t15”) [58]. Thirdly, all genomes were translated into all possible amino acid sequences of length 15 or more and scanned for homologs of entries in the Pfam database of protein families using hmmsearch (v3.1dev and the parameter “–cut_ga”) [41], [42].

### Identification of novel RNAs

From the mapped RNA-seq data, potential novel RNA genes (designated RNAs of Unknown Function, or RUFs) were picked manually by locating regions in the genomes that showed high levels of expression without overlapping annotated protein coding or RNA genes. Only RUFs of lengths 50 to 400 nucleotides were included, yielding a total of 844 RUFs in Bacteria and 78 RUFs in Archaea.

### Homology search and structure prediction

Homologs of the identified RUFs were found in all the downloaded genomes using nhmmer [59] in an iterative fashion: First, the RUF sequence alone was used in the scan; then, all hits with E-value 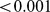 were included and a HMM built. This was iterated 5 times. The alignments from the RUF homology search were analyzed further by investigating the potential for secondary structure formation using RNAz [60] and alifoldz [61]. Protein coding potential of the RUFs was assessed using RNAcode [62]. Overlaps between potential RUF homologs in other strains/species and all the annotations in the respective genomes were also assessed.

### Comparative expression and conservation analysis

For each strain, the available RNA-seq datasets were pooled and a list was created of transcripts showing expression in that strain in at least one experiment (defined as a transcript having a median depth of at least 10 reads in any experiment). A RUF homolog was defined as being expressed if the median read depth of the homologous region was at least 10X. Transcripts were ranked for each strain based on median expression (i.e. the most highly expressed transcript will have rank 1), which makes relative comparison across strains and datasets possible. The final set comprises 452 different Rfam families, 922 different RUFs, and 7249 different Pfam domains.

For comparative analysis, if a gene was found to be expressed in more than one strain/species, the minimum rank was used (i.e. showing the relatively most abundant expression of the gene). This ensures that transcripts that are always low abundance will remain low abundance, whereas genes that are highly abundant in at least one of the sampled time points and conditions will be treated as such. The ranking is used as a measure of expression.

“Family conservation” is based on SSU rRNA alignments of all Bacteria and Archaea, respectively. For each genome, the best hit to the Rfam model of SSU rRNA was extracted (RF00177 for Bacteria and RF01959 for Archaea). The sequences were aligned to the model using cmalign [57]. Finally, a distance matrix was calculated using dnadist [63] with the F84 model [64], [65] which allows for different transition/transversion rates and for different nucleotide frequencies. The pairwise strain/species distances produced in this manner estimate the total branch length between any pair of strains/species. For any gene found in two or more strains/species, the maximum pairwise distance is used as the conservation score. Upper and lower quartiles of the distributions are used to define sets of high, medium and low expression and conservation, respectively. (Expression, upper quartile: 204. Expression, lower quartile: 1660. Conservation, upper quartile: 0.478. Conservation, lower quartile: 0.267).

### Quality control of RNA-seq datasets

We ranked datasets based on the following quality control metrics (values reported in Table S3).

#### Strand correlation

We calculated correlation between the reads on the two strands. If the dataset is unstranded, we expect a correlation close to 1.

#### Expression of core genes

We defined a set of 40 core protein-coding genes based on [66], [67] and 16 noncoding RNA genes (the union of tRNA, RNaseP, tmRNA, SRP, 6S and rRNA RNA families) [22], [35]. If the median read depth is greater than 10X, we defined the gene as expressed. For each dataset, we report the fraction of the core genes that are expressed.

#### Coverage

We calculated coverage as the fraction of the genome covered by at least 10 mapped reads.

#### Fraction mapped reads

For each dataset, we ascertained the fraction of mapped reads.

#### Concordance

To measure how well a given RNA-seq dataset corresponds to the annotated genes in a genome, we developed a concordance metric. For this, we define true positives (TP) to be the number of annotated positions that are expressed; false positives (FP) to be the number of unannotated positions that are expressed; true negatives (TN) to be the number of unannotated positions that are not expressed; and false negatives (FN) to be the number of annotated positions that are not expressed. Note, not all annotated genes are expected to be expressed, and not all unannotated positions are false. Therefore, we calculate the positive predictive value (PPV): 
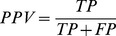



This measures the fraction of expressed positions that are annotated. We also calculate the fraction of the genome that is annotated: 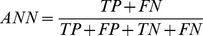



To make the PPV more robust, our final concordance metric normalizes PPV by ANN.

## Supporting Information

Table S1 The Pfam, Rfam and RUF identifiers for each entry corresponding to Figure 1.
(XLS)

Table S2 Strain/species names, genome accessions, RNA-seq data sources, Pubmed IDs, sequencing platform and notes for each dataset used for this study.
(XLS)

Table S3 Quality control measures computed for each RNA-seq dataset used in this study. The values are defined in detail in the Methods section.
(XLS)
